# Determining factors of university students' binge-watching attitudes

**DOI:** 10.1016/j.heliyon.2024.e39642

**Published:** 2024-10-21

**Authors:** Murat Aytas, Ismail Berkay Topatan

**Affiliations:** a Department of Radio, Television and Cinema, Selcuk University, Konya, Turkey; b Department of Radio, Television and Cinema, Institute of Social Sciences, Selcuk University, Konya, Turkey

**Keywords:** Binge-watching, Watching motivations, Video on demand, Digital video platforms, Serials

## Abstract

Binge-watching (BW), defined as watching multiple episodes of a series or program in a single sitting, is recognized as a new and widespread form of viewing, especially with online streaming services. This study investigates the factors determining university students' binge-watching attitudes and the relationship of these factors with demographic characteristics. Data were collected using a survey adapted from the "Watching TV Series Motives Questionnaire (WTSMQ)" and the "Binge-Watching Engagement and Symptoms Questionnaire (BWESQ)" developed by Flayelle and colleagues (2019). The survey was conducted online with the participation of 636 university students to evaluate participants' motivations for watching internet series, their BW attitudes, and the relationship of these behaviors with demographic factors such as age, gender, and education level. The results demonstrate that students' BW behavior is strongly associated with positive motivations such as ease of access, entertainment, and social interaction. Specifically, a significant positive correlation between entertainment motivation and BW behavior was found (r = .240, p > .01). Additionally, it was determined that female students exhibit significantly higher BW behavior compared to male students, and BW attitudes/behaviors decrease with increasing age and education level. Participants who have never worked show more BW behavior compared to those who have worked or are currently working.

## 1 Introduction

With the dominance of a culture of convergence, technological advancements and innovations in the media field provide opportunities that fundamentally change the culture of watching television and television series. The tools and applications provided by internet technology allow viewers to follow visual content through various means, supporting the development of new viewing experiences. One of these experiences is “binge-watching” (BW), defined as viewing multiple episodes of serialized video content in a single sitting at an individual's own pace and time [1]. Streaming services prioritize viewer preferences and have emerged as a new and widespread viewing experience, making BW almost a norm [[2], [3], [4], [5]].

Research shows that platforms like Netflix and Disney + allow viewers to plan their entertainment schedules, creating a sense of autonomy that leads to enjoyment and well-being [2,[6], [7], [8], [9]]. However, conflicts between entertainment consumption, daily tasks, and a loss of control can lead to adverse outcomes. While some studies suggest that watching series can be enjoyable and increase personal well-being [2] excessive viewing may negatively affect social relationships and physical and psychological health [10,11], cause concentration problems [12] and worsen mental health issues [13]. Studies have shown that excessive watching is associated with regret, distress, and unhappiness [[14], [15], [16]] and can lead to adverse outcomes such as loss of control, social problems, and neglect of duties [17]. Furthermore, excessive viewing has been associated with decreases in exercise and healthy eating [18] and is considered a type of addictive behavior [[19], [20], [21]]. Previous studies discuss the motivations behind behaviors like BW and how they affect viewers' psychological and physical states, and they raise concerns about these issues. When viewers decide to watch multiple online TV episodes, curiosity, and excitement push aside pragmatic thinking, leading to misconceptions about the availability of time and its impact on daily routines [22]. This situation, especially with the uninterrupted or unlimited access to online screen time among adolescents and young adults, necessitates the exploration of the phenomenon of consecutive online series and movie watching. In this context, Sun and Chang [23] identify the prevalence of this behavior among young adults and the concerns it creates for physiological, mental, and social well-being as an "urgent issue" that requires research. Previous research encompasses various views on defining BW behavior [5,24,25] and lacks sufficient dimensions, especially regarding inclusivity. Although many studies have been conducted in the USA and various European countries, researchers have reported different findings on the motivations and outcomes of consecutive watching in societies with varying levels of economic development and cultures. For instance, Merrill et al. (2019) found that people in developing countries have stronger associations with stress and sleep problems due to BW than those in developed countries. Reviews of related literature specifically highlight the need to enrich the data on the subject with studies to be conducted in different cultural contexts [24,[26], [27], [28], [29], [30]].

The current study aims to contribute to understanding BW by relying on a field survey conducted in Turkey, where research is still quite limited. This study specifically seeks to measure the motivations affecting students' BW behaviors and the relationship of this behavior with various factors such as age, education level, employment status, and income level among university students who watch series on online digital streaming platforms.

However, the current study's conceptualization of "binge-watching" (BW) is insufficient and does not clearly distinguish between its different contexts. BW is generally defined as watching multiple episodes of a series or program in a single sitting [21]. Yet, this concept varies significantly in different contexts and its impacts on viewers can be diverse. Granow et al. [2] note that BW can create a sense of autonomy and enhance well-being, whereas Steins-Loeber et al. [17] highlight negative psychological effects like depression, anxiety, and sleep disorders. The definition of BW should consider factors such as frequency, duration, and intent of viewing [31], as well as social and individual contexts [32,33]. Addressing these differences is crucial for a comprehensive understanding of BW. Future research should include more detailed definitions and measurement methods to better capture the complexity of BW behaviors and their impacts [2,17,21,[31], [32], [33]]. Since digital streaming platforms like Netflix started operating in Turkey in 2016, research in this area has been limited in both number and scope. A significant portion of the conducted research focuses on topics such as the structure of the Netflix interface, how viewers interact with these environments, and their contribution to the transformation of the broadcasting field [[34], [35], [36]]. In some of these studies [[37], [38], [39]] findings aim to understand the transformation of viewers' consumption habits, motivations for BW, and outcomes. Considering the concept's popularity, researchers have found that a significant portion of research on BW was conducted with quite limited participants, that findings from different studies were contradictory, and that they did not adequately examine how BW behavior changes according to various demographic determinants. To enhance the comprehensiveness of the review, recent studies and theoretical frameworks that specifically address the factors influencing binge-watching attitudes and motivations in this demographic group have been included [[40], [41], [42], [43]]. The current literature on binge-watching (BW) behavior highlights its popularity and complexity, especially among young adults and university students, driven by platforms like Netflix and Disney+. Studies consistently define BW as watching multiple episodes in a single sitting, with motivations including entertainment, relaxation, social interaction, and escapism. However, there are inconsistencies in how these motivations are emphasized across different demographics and cultures. Research extensively documents the negative psychological and physical impacts of excessive BW, such as increased risks of depression, anxiety, and sleep disorders, highlighting its potential as an addictive behavior. There are significant sociocultural variations in the effects and motivations behind BW, with stronger associations to stress and sleep problems in developing countries compared to developed ones, underscoring the need for more culturally inclusive research. Addressing these gaps, the study is anticipated to contribute to the literature with its approach to understanding BW behavior and motivations, considering the sample's breadth and the participants' demographic characteristics.

## 2 Literature review

The roots of the binge-watching (BW) phenomenon lie in watching and entertainment technologies like DVDs and VCRs [44]. However, the transformation of this watching behavior into almost a norm occurred with the proliferation of online television service providers. This form of viewing, combined with personalized recommendations, has become a model for releasing and consuming new content on VOD (Video on Demand) [[45], [46], [47], [48]]. The techniques of streaming services like Netflix and Amazon Video, which encourage consecutive viewing, have made the viewing style more accessible and flexible [24]) and have marked a significant departure from previous media consumption forms [32,49]. Freed from the constraints of linear broadcast schedules, viewers have had the opportunity to create a more personalized viewing schedule by programming and revisiting content according to their own time, immersing themselves in a deeper relationship with the text ([49,50]. This viewing design, based on the viewer's active participation, has changed consumers' viewing habits and resulted in the watching of multiple episodes in a compressed timeframe [29,30,32,51].

There are various approaches in the literature on defining binge-watching (BW). While some studies consider the number of episodes watched [1,15,16,24,31,33,[52], [53], [54], [55]], others emphasize the importance of the time spent in front of the screen [56], the frequency of this behavior [12,51], or adopt a multi-perspective approach that encompasses all or some of these factors [3,25,57]. The effect of watching the same number of episodes of programs of different lengths remains a matter of debate [1]. Moreover, the frequency of the BW experience poses a problem when defining and measuring the concept. It may be misleading to categorize the act of watching a certain number of episodes or an entire season in a short period in the same category as this action turning into a behavior repeated on different days. In this context, the literature generally accepts [1,15,24,46,52,55,57,58] and uses the concept to name the act of watching multiple consecutive episodes of a TV show, encompassing all forms of excessive viewing.

The association of series watching with terms like "binge eating," used to describe excessive behaviors such as overeating or drinking to a self-harming extent [45], frames it as an embarrassing behavior implying a lack of self-control. Various studies focus on the adverse effects on physical, psychological, and social well-being associated with a lack of self-control and the inability to control watching, including procrastination behavior, lethargy, loss of fitness [59], stress, sleep problems [13] depression, loneliness [60], a sedentary lifestyle, disordered eating behaviors [10], impairment in daily functionality, or emotional distress [7,[17], [18], [19],^33^,^54^].

However, various studies provide evidence about the heterogeneous nature of binge-watching (BW). Indeed, research has found that binge-watching can also be a typical situation where positive emotions and need satisfaction are met without issues, or content is watched in series due to motivations prioritizing positive affect [61]. This highlights the complexity of BW, indicating that it can have negative and positive dimensions depending on individual motivations and outcomes. Research highlights two main perspectives in understanding binge-watching (BW). The first approach associates BW with positive emotions such as entertainment and making use of leisure time [2,31,62]and with social and cognitive motivations [32,55]. The second perspective emphasizes the adverse outcomes, particularly of excessive BW, associating it with issues like depression, anxiety, sleep problems, and mental disorders [7,13,17,19] There are also findings suggesting that BW can be a form of addiction [45,63]. However, it has been shown that the likelihood of adverse effects is lower in cases where viewing is intentional or planned [20,63,64] and that, compared to other forms of addiction, BW does not fully meet the criteria for behavioral addiction [5,63]. The research questions (RQ 1 to RQ 4) were formulated based on gaps identified in the existing literature and the need to understand the factors influencing binge-watching (BW) behaviors among university students. In this context, this study aims to understand the BW behaviors of students watching series on online streaming platforms, the motivations triggering these behaviors, how watching is experienced, and how it may vary according to demographic characteristics, thereby determining the following research questions.

• RQ 1: How are the motivations for watching internet series distributed among participants?

Previous studies have identified a variety of motivations for binge-watching, such as ease of access, entertainment, relaxation, and social interaction [[2], [3], [4],^65^]. However, there is a need for a detailed examination of these motivations specifically among university students, who extensively use digital streaming platforms [65]. Understanding these motivations can help in tailoring content and improving user experiences on streaming platforms [5,11].

• RQ 2: How are participants' attitudes/behaviors towards binge-watching (BW) distributed?

While binge-watching behaviors have been widely studied, there is a lack of detailed data on how these behaviors are distributed among university students in different contexts [12,65]. Previous research has primarily focused on general populations or specific age groups, but the specific patterns and attitudes towards BW in university students remain underexplored. This research question aims to fill that gap by providing a comprehensive overview of BW behaviors within this demographic [2,15].

• RQ 3: Is there a significant relationship between participants' motivations for watching internet series and their attitudes/behaviors towards BW?

The relationship between viewing motivations and BW behaviors has been suggested in various studies, but empirical evidence on this relationship is inconsistent and sometimes contradictory [16,17]. By investigating this relationship among university students, this study aims to provide clearer insights into how different motivations influence BW behaviors, contributing to the broader understanding of digital content consumption [2,15].

• RQ 4: Do participants' attitudes/behaviors towards BW significantly differ according to their demographic characteristics?

Demographic factors such as age, gender, education level, and employment status have been shown to influence media consumption behaviors, including binge-watching [4,10,12]. However, the specific impact of these factors on BW behaviors among university students has not been thoroughly investigated. This research question aims to explore how these demographic variables affect BW attitudes and behaviors, providing valuable data for content providers and policymakers [1,15].Addressing these questions can provide insights into the underlying factors that influence binge-watching behaviors and the impact of demographic variables on these behaviors, contributing to a deeper understanding of the dynamics of digital content consumption.

## 3 Method

### 3.1 Design and participants

This cross-sectional study investigates the relationship between motivations for watching series online and attitudes and behaviors towards binge-watching (BW), as well as the differences based on demographic characteristics among university students in Konya. Considering the Higher Education Council (YOK) 2023 data, the number of university students aged 18 and over in Konya has been determined to be 127,272. Based on calculations, with a 95 % confidence interval and a 3 % margin of error considered, it has been determined that a sample size of at least 500 individuals is required to represent this research population. The study participants consisted of university students registered at Selcuk University who were actively pursuing their education, were over 18, and had no additional diseases. Participation in the study was based on voluntariness, and all participants completed all or at least the mandatory fields of the survey questions. ERASMUS students with insufficient Turkish language proficiency that could prevent a correct understanding of the informed consent form and the survey, students with disabilities or metabolic disorders, and students under 18 were excluded. Considering potential issues with response rates to the online survey created between March 7, 2021, and May 10, 2022, researchers reached a total of 744 individuals, of which 636 (85.4 %) agreed to participate in the study and filled out the survey to be included in the sample. To ensure the anonymity of the data, each participant was assigned a number, and the data was recorded anonymously. The data was processed according to the principles of the Helsinki Declaration. The study was approved by the Selcuk University Research Ethics Committee (E.100851).

### 3.2 Pilot test

A pilot test was conducted to ensure the validity and reliability of the survey and to confirm that participants clearly understood the questions. For the pilot test, 50 university students who were not part of the main study were selected. These students completed the survey online, and feedback was requested regarding the clarity of the questions. Based on the feedback received, the survey questions were reviewed, and necessary adjustments were made. This process was carried out to ensure that the final version of the survey would be easily understood by participants and that accurate responses would be provided.

### 3.3 Instruments

The study's questionnaire consists of three parts based on the information it seeks. The questions were prepared by researchers utilizing the "Watching TV Series Motives Questionnaire (WTSMQ)" and "Binge-Watching Engagement and Symptoms Questionnaire (BWESQ)" scales developed by Flayelle and colleagues (2019). The WTSMQ and BWESQ scales are mtwo essential tools that measure series-watching behavior and motivations and have been used in many studies in this field [33,66]. The questionnaire, comprising a total of twenty-seven questions, includes ten questions in the first part aimed at measuring participants' motivations for watching internet series, twelve questions in the second part aimed at assessing the participants' attitudes/behaviors towards BW, and five questions in the third part aimed at determining the individual characteristics of the participants (demographic variables). Participants responded to items on a five-point Likert scale ranging from 1 (strongly disagree) to 5 (strongly agree). The total reliability of the scale was obtained with a Cronbach's alpha value of .738. The validity of the study was ensured through face validity. The researchers prepared the questions using a literature review and relevant research findings. The overall average and reliability values for the sub-scales of the questionnaire were calculated. The reliability value of the internet series watching motivation scale was measured as alpha = .806, indicating consistently good internal consistency. The reliability value of the binge-watching (BW) attitude scale was measured as alpha = .739, which shows acceptable internal consistency. These reliability coefficients demonstrate that both scales are reliable for measuring their intended constructs.

The data obtained within the scope of the research were subjected to different analyses to answer the research questions. Descriptive statistics tools (frequency, lowest, highest, arithmetic mean, median, and mode) were used to determine how the distribution of participants' motivations for watching internet series appears. This approach summarizes wide data ranges, such as participants' watching motivations from demographically diverse groups, and quickly identifies fundamental trends and patterns. Descriptive statistical tools are crucial for summarizing large data sets and making them meaningful. These tools provide information about data distribution by calculating values such as mean, median, mode, standard deviation, frequency, and percentage [67]. Using descriptive statistics tools in other studies [21,54] that utilized measurement instruments for researching the conceptualization of BW supports this methodology. These tools help make large datasets manageable and understandable, allowing for a more straightforward interpretation of the data's underlying distribution and central tendencies. In a further evaluation of participants' motivations for watching internet series, these motivations were grouped into four dimensions: entertainment, relaxation/escape, social interaction and social information gathering, and each dimension was evaluated through central tendency statistics (mean, median). Inspired by the five-point Likert scale, where the level of agreement ranges from a minimum of 1 to a maximum of 5, a total participation index was created, and ratings were scaled starting from 1 in a range of 4/5 = .80. The classifications were as follows: 1.00–1.80 = very low; 1.81–2.60 = low; 2.61–3.40 = medium; 3.41–4.20 = high; 4.21–5.00 = very high.

Regarding the study's second research question, the data obtained on participants' attitudes and behaviors towards binge-watching (BW) were presented through central tendency statistics (mean, median) to highlight the general trend in BW behaviors and allow for comparison of variations across different groups. Researchers used central tendency statistics to understand this complex behavior better and identify factors associated with BW (Starosta et al., 2020). A second evaluation was conducted to reach more in-depth conclusions regarding participants' attitudes towards BW. All attitude items were converted into a single index, and an attempt was made to obtain a more holistic evaluation by calculating the central tendency statistics of this index. This classification also drew inspiration from the five-point Likert scale, with ratings starting from 1 in a range of 4/5 = .80, based on a total participation index. The classifications were as follows: 1.00–1.80 = very low; 1.81–2.60 = low; 2.61–3.40 = medium; 3.41–4.20 = high; 4.21–5.00 = very high. A Pearson correlation analysis was conducted to determine the direction and magnitude of the possible relationship between participants' motivations for watching internet series and their attitudes/behaviors towards binge-watching (BW). Many studies that have utilized Pearson correlation analysis to understand BW behavior [21,32,68] have provided insights into how strong the impact of various factors on BW behaviors is and whether this relationship is positive or negative. The Pearson correlation analysis is an effective statistical tool for measuring the degree of linear relationship between two variables, offering valuable insights into how closely related the motivations for watching series online are to the attitudes and behaviors towards BW, thus contributing to a deeper understanding of the binge-watching phenomenon.

In addressing the fourth research question of the study, tools such as one-way analysis of variance (ANOVA), independent sample *t*-test, and correlation analysis were utilized to determine whether participants' attitudes/behaviors towards binge-watching (BW) significantly differed according to their demographic characteristics. An independent samples *t*-test was applied to test the hypothesis predicting a significant difference in BW attitude levels based on gender. Researchers used this analysis technique to compare the mean scores of the BW attitude index between two independent sample categories, such as the gender variable, when it was necessary to compare interval-level measurements. To test the hypotheses predicting significant differences in participants' BW attitude levels according to age, years of education, employment status, monthly spending amount, platform used, and frequency of series watching, researchers applied a one-way analysis of variance (ANOVA). ANOVA was preferred in these analyses because there were more than two independent sample categories. It was necessary to compare the mean scores of the BW attitude index at an interval measurement level. These statistical analyses help understand the influence of various demographic factors on binge-watching behaviors and attitudes, providing a comprehensive understanding of how these factors relate to BW engagement.

## 4 Results

### 4.1 Descriptive findings on motivations for watching TV series on the internet

Based on the demographic data obtained from the study, the average age of the participants is 22. Of the participants, 53.8 % are male and 46.2 % are female. 33.8 % of the participants have completed a master's degree. Currently, 49.5 % of the participants are actively working. Among the participants, 13.4 % have never worked before, 26.3 % are not working now, and 10.8 % have previously worked part-time. Table 1 presents detailed demographic data of the participants. This distribution of demographic characteristics among participants provides a comprehensive overview that can be instrumental in understanding the diverse perspectives and behaviors toward binge-watching across different demographic segments.

**Table 1:** Distribution of participants' differences.

	Group	Frequency	Percentage
Age	18–19	40	6.3
	20–21	90	14.2
	22–23	116	18.2
	24–25	168	26.4
	26 +	222	34.9
Education Status	Prep Class	17	2.7
	First-year	25	3.9
	Second-year	96	15.1
	Third year	28	4.4
	Fourth-year	121	19.0
	Extended student	82	12.9
	Master's Degree	215	33.8
	PhD	52	8.2
Employment Status	Never worked before	85	13.4
	I am not currently working	167	26.3
	I have worked part-time before	69	10.8
	I am currently actively working	315	49.5
Gender	Male	342	53.8
	Female	294	46.2

The descriptive statistics analysis of the responses given by participants regarding their motivations for watching series online reveals that they find positive motivations such as accessibility, entertainment, and information seeking more determinant in their decision to watch internet series. Examining the analysis results in Table 2, we see that participants gave the highest positive responses to motivations related to accessibility, entertainment, and information seeking. Following these are the responses given to relaxation and social interaction motivations. On the other hand, the items that participants agreed with the least were associated with harmful content motivations such as depressive mood and escapism.

**Table 2:** Internet Series Viewing Motivations: entertainment, relaxation/escape, social information, and social interaction dimensions Central Tendency Statistics.

	N	Lowest	Highest	Avg.	S.deviation
Watching dramas entertains me.	636	1	5	3.90	.945
I have nothing better to do than watch TV series.	636	1	5	1.84	1.143
**Entertainment Dimension General:**	**636**	**1.00**	**5.00**	**2.87**	**.814**
Internet dramas come quickly to me	636	1	5	4.27	.958
Watching web series relaxes me	636	1	5	3.68	1.161
Thanks to the web series, I forget my daily life troubles	636	1	5	2.89	1.275
**Relaxation/Escape Dimension General**	**636**	**1.33**	**5.00**	**3.61**	**.744**
Thanks to the web series, I understand different lives and people.	636	1	5	3.81	1.089
TV series provide me with ideas about current issues and the agenda.	636	1	5	3.09	1.250
TV series help me direct my daily life and thoughts.	636	1	5	2.37	1.196
**Social Informatization Dimension General:**	**636**	**1.00**	**5.00**	**3.09**	**.744**
Internet series enable me to establish common bonds with people.	636	1	5	3.18	1.195
Internet series prevent me from feeling lonely.	636	1	5	2.93	1.281
**Social Interaction Dimension General:**	**636**	**1.00**	**5.00**	**3.05**	**.962**

Based on the results, the average index for the entertainment dimension among participants is 2.87, indicating that their importance on the entertainment aspect is considered at a "medium" level. The average of the relaxation/escape dimension index is 3.61, which shows that participants attribute a "high" level of importance to the relaxation/escape aspect. According to the results of the central tendency statistics, the social information gathering dimension index average is 3.09, leading to the conclusion that the importance placed on social information gathering by participants is at a "medium" level. The average social interaction dimension index is 3.05, indicating that participants place a "medium" level of importance on social interaction. These findings highlight the varied motivations behind participants' engagement with internet series watching, with relaxation/escape emerging as a particularly significant motivator compared to entertainment, social information gathering, and social interaction, which are considered of medium importance. This differentiation in motivational importance can inform content creators and platforms about viewer preferences and potentially guide the development of more engaging and targeted content.

### 4.2 Descriptive findings on BW attitudes and behaviors

The findings obtained regarding participants' attitudes and behaviors towards binge-watching (BW), as revealed through central tendency statistics, are presented in Table 3. Upon examining the analysis results, it was determined that participants responded most positively to the characteristics of continuity and immersion. The opportunity to watch multiple episodes or even a complete season in one sitting without interruption by advertisements has a significant impact on participants' BW behaviors. Furthermore, participants have also indicated a high preference for being more sedentary than usual while watching. These insights suggest that the seamless viewing experience and immersive content consumption without disrupting advertisements are vital factors that enhance the binge-watching experience. Additionally, the tendency to remain sedentary during binge-watching sessions reflects a significant aspect of viewer behavior, emphasizing the importance of comfort and uninterrupted engagement in the binge-watching phenomenon.

**Table 3:** Central tendency statistics for binge-watching attitudes and behaviors.

	N	Lowest	Highest	Avg.	S. deviation
Serial content (series/movies) I watch online is more appealing than content interrupted by commercial breaks on TV.	636	1	5	4.29	1.024
Watching one season or multiple episodes at a time immerses me in the story.	636	1	5	4.23	.999
I am less mobile than usual during my viewing activity.	636	1	5	3.94	1.024
I engage in more viewing activities in my free time at home.	636	1	5	3.93	1.098
When the content I watch is interrupted by advertisements, my interest in the content I watch decreases.	636	1	5	3.82	1.209
I think binge-watching is related to popular culture.	636	1	5	3.62	1.249
I do not want to be interested in something else until the episode/season I am watching ends.	636	1	5	3.30	1.149
During my binge-watching activity, I consume more junk food/food/drink than usual.	636	1	5	3.17	1.289
I do not leave the screen unless there is a fundamental reason.	636	1	5	3.13	1.215
I am not satisfied with my weight.	636	1	5	3.03	1.545
I complain of back/head/neck pain in my body in general.	636	1	5	2.89	1.248
When I do a long-term viewing activity, I experience short-term regret.	636	1	5	2.86	1.290
**Valid N (listwise)**	**636**				

On the other hand, the items that participants showed the lowest level of agreement with are related to weight problems, physical discomfort that could arise from prolonged viewing activity, and feelings of regret. These findings indicate that while participants highly value binge-watching's uninterrupted and immersive aspects, they are less concerned with potential adverse physical health outcomes or feelings of regret associated with prolonged sedentary behavior during binge-watching sessions. Additionally, a second evaluation was conducted in the study to achieve more in-depth results regarding participants' attitudes towards binge-watching (BW). All attitude items were consolidated into a single index, and an attempt was made to obtain a more detailed evaluation by calculating the central tendency statistics of this index. The central tendency statistics related to BW attitudes are presented in Table 4. This comprehensive approach to analyzing attitudes towards BW helps to highlight the complex and multifaceted nature of binge-watching behavior, including its appealing aspects and potential areas of concern among viewers. By examining the overall attitude index, researchers can gain insights into the general disposition of participants towards BW, encompassing a range of factors from enjoyment and convenience to health and psychological impacts.

**Table 4:** Binge watching attitude/behavior index central tendency statistics.

	N	Lowest	Highest	Avg.	S. deviation
Binge Watching	636	1.17	4.75	3.5178	.61179
**Valid N (listwise)**	**636**				

According to the results, the average index for participants' attitudes/behaviors towards binge-watching (BW) is 3.51, indicating that the level of participants' attitudes/behaviors towards BW can be considered "high." The results of the Pearson correlation analysis to inquire about the possible relationship between participants' motivations for watching internet series and their attitudes/behaviors towards BW are provided in Table 5. According to these results, there is a significant positive relationship between participants' BW attitude and their motivations for watching internet series such as social interaction (r = .492, p > .01), social information gathering (r = .469, p > .01), relaxation/escape (r = .332, p > .01), and entertainment (r = .240, p > .01).

**Table 5:** Correlation analysis results of binge-watching attitude and internet series viewing motivations.

		Relaxation/Escape	Social Interaction	Entertainment	Social Knowledge
Bing Watching Attitude	**r**	.332∗∗	.492∗∗	.240∗∗	.469∗∗
	**Sig.**	.000	.000	.000	.000
	**N**	.636	.636	.636	.636

These findings suggest that the more participants are motivated by social interaction, social information gathering, relaxation/escape, and entertainment, the more positive their attitudes and behaviors toward binge-watching tend to be. This highlights the importance of these motivational factors in influencing binge-watching behaviors, suggesting that interventions or content strategies aiming to address binge-watching might consider these motivational aspects to better align with viewer preferences and behaviors.

### 4.3 BW attitude and demographic characteristics of participants

In this study section, hypotheses predicting that the levels of attitudes/behaviors towards binge-watching (BW) would differ according to participants' demographic characteristics were tested through relevant analyses. According to the analysis results presented in Table 6, the BW attitude levels of student participants significantly differ based on their gender (t = 3.675, p < .05). Female participants (‾X = 3.61) have significantly higher BW attitude levels compared to male participants (‾X = 3.43).

**Table 6:** Binge-Watching Attitude Index and Gender-Independent Sample *t*-Test Results.

	Gender	N	Avg.	*t*-Test	Sig.
**Binge Watching Attitude Index**	Female	294	3.61	3.675	**.000**
	Male	342	3.43		

According to the findings of the research, participants' attitudes towards binge-watching (BW) significantly differ based on their ages (F = 3.115, p <. 05). The analysis results detailed in Table 7 indicate a trend of decreasing BW behavior as age increases. This trend suggests that younger participants are more inclined towards binge-watching than older participants. The decrease in binge-watching behavior with age could be influenced by various factors, such as changes in lifestyle, responsibilities, time availability, or even shifts in media consumption preferences.

**Table 7:** Bing watching attitude index and age ANOVA results.

	Age	N	Avg.	F Test	Sig.
**Binge Watching Attitude Index**	18–19	40	3.76	3.115	**.015**
	20–21	90	3.51		
	22–23	116	3.61		
	24–25	168	3.45		
	26 +	222	3.47		

The Tamhane test was applied to identify which age groups have different attitudes towards binge-watching (BW). According to the multiple comparison table obtained, the BW attitude levels of participants aged 18–19 (‾X = 3.76) are significantly higher compared to those in the age groups of 20–21 (‾X = 3.51), 24–25 (‾X = 3.45), and 26 and over (‾X = 3.47).

Furthermore, analysis results indicate that participants' BW attitude levels significantly differ based on their years of study (F = 3.184, p <. 005; Table 8). The Tamhane test was also applied to determine in which study year groups the BW attitude levels differ. According to the multiple comparison table obtained, the BW attitude levels of participants in the preparatory class (‾X = 3.81) are significantly higher than those of the master's (‾X = 3.48) and doctoral (‾X = 3.47) groups; first-year students' (‾X = 3.90) attitude levels are significantly higher compared to the second (‾X = 3.45), fourth (‾X = 3.47), extended (‾X = 3.49), master's (‾X = 3.48), and doctoral (‾X = 3.47) groups. Similarly, the BW attitude levels of third-year students (‾X = 3.78) are significantly higher than those of fourth-year (‾X = 3.47), extended (‾X = 3.49), master's (‾X = 3.48), and doctoral (‾X = 3.47) groups.

**Table 8:** Bing watching attitude index and year of study ANOVA results.

	Year (Class)/Program	N	Avg.	F Test	Sig.
**Binge Watching Attitude Index**	Prep Class	17	3.81	3.184	**.003**
	First-year	25	3.90		
	Second-year	96	3.45		
	Third-year	28	3.78		
	Fourth-year	121	3.47		
	Extended student	82	3.49		
	Master's Degree	215	3.48		
	PhD	52	3.47		

According to the research findings, participants' attitudes towards binge-watching (BW) also significantly differ based on their employment status (F = 17.530, p <. 001; see Table 9). The Tamhane test, applied to determine between which work status groups the BW attitude levels differ, resulted in a multiple comparison table indicating that participants who have previously worked part-time have significantly higher BW attitude levels (‾X = 4.00) compared to those who have never worked (‾X = 3.48), are currently not working (‾X = 3.47), and are currently working full-time (‾X = 3.44).

**Table 9:** Bing watching attitude index and employment status ANOVA results.

	Employment Status	N	Avg	F Test	Sig.
**Binge Watching Attitude Index**	Never worked before	85	3.48	17.530	**.000**
	I am not currently working	167	3.47		
	I have worked part-time before	69	4.00		
	I am currently actively working	315	3.44		

According to the analysis results detailed in Table 10, participants' attitudes towards binge-watching (BW) do not significantly differ based on their monthly spending amounts (F = .947, p >. 05).

**Table 10:** Bing watching attitude index and monthly expenditure ANOVA results.

	Monthly Expenditure Amount (Turkish Liras)	N	Avg	F Test	Sig.
**Binge Watching Attitude Index**	50–249	45	3.38	.947	.412
	250–499	59	3.59		
	500–749	126	3.51		
	750 +	406	3.52		

According to the analysis results, participants' attitudes towards binge-watching (BW) significantly differ based on their preferred platform (F = 9.335, p <. 001; see Table 11). The Tamhane test was applied to determine which BW attitude levels differ among platform groups. According to the multiple comparison tables obtained, participants who prefer Puhu TV (‾X = 3.55) have significantly higher BW attitude levels compared to those who prefer Netflix (‾X = 3.19) and Amazon Prime (‾X = 3.44).

**Table 11:** Bing watching attitude index and preferred platform ANOVA results.

	Preferred Platform	N	Avg.	F Testi	Sig.
**Binge Watching Attitude Index**	Netflix	24	3.19	9.335	**.000**
	Blu TV	23	3.60		
	Puhu TV	554	3.55		
	Amazon Prime	35	3.08		

Finally, according to the analysis results detailed in Table 12, participants' attitudes towards binge-watching (BW) significantly differ based on their frequency of series-watching (F = 28.101, p <. 001). The Tamhane test was applied to identify which frequency groups the BW attitude levels differ. According to the multiple comparison tables obtained, participants who watch series rarely (‾X = 3.17), occasionally (‾X = 3.39), frequently (‾X = 3.59), and very frequently (‾X = 3.62) have significantly higher BW attitude levels compared to those who never watch (‾X = 2.03). Moreover, participants who watch series frequently (‾X = 3.59) and very frequently (‾X = 3.62) have significantly higher BW attitude levels compared to those who never watch (‾X = 2.03), rarely watch (‾X = 3.17), and occasionally watch (‾X = 3.39).

**Table 12:** Bing watching attitude index and series viewing frequency ANOVA results.

	Frequency of Watching Series	N	Avg.	F Test	Sig.
**Binge Watching Attitude Index**	I Never Watch	12	2.03	28.101	**.000**
	I Rarely Watch	35	3.17		
	I watch occasionally	103	3.39		
	Frequent Viewers	351	3.59		
	I Watch Very Often	135	3.62		

## 5 Discussion

This study aimed to explore the factors influencing university students' binge-watching (BW) behaviors. The first research question examined the distribution of motivations for watching internet series. Findings showed that positive motivations such as ease of access, entertainment, and social interaction were dominant. Relaxation/escape was the most significant motivation, supporting the hypothesis that students prioritize these positive factors over escapism or loneliness.

Next, the study analyzed participants' binge-watching attitudes and behaviors. Results indicated that most students exhibited high levels of BW, with continuity and immersion being key factors. The uninterrupted and ad-free experience provided by digital platforms was especially important, confirming the hypothesis that seamless viewing drives BW behaviors.

The relationship between motivations for watching series and BW behaviors was also explored. Pearson correlation analysis showed a significant positive link between motivations like entertainment, social interaction, and relaxation/escape, and BW attitudes. This supports the hypothesis that higher motivation levels are associated with more frequent binge-watching.

Finally, the study investigated demographic differences in BW behavior. Results indicated that female students exhibited higher BW behavior than males, and BW tendencies decreased with age and education level. Additionally, students who had never worked showed higher BW behavior, confirming that demographic factors significantly influence BW tendencies.

In this study, we aimed to investigate the binge-watching (BW) behaviors of university students and how demographic characteristics influence these behaviors. The findings reveal several important contributions and identify gaps in the current research. Table 13 summarizes these contributions and research gaps.

**Table 13:** Contributions and research gaps in the study of binge-watching (BW) behavior among university students.

Research Area	Contributions	Gaps
**Definition and Motivations of Binge-Watching (BW)**	Identified key motivations affecting students' BW behavior: ease of access, entertainment, and social interaction.	Detailed examination of BW behaviors in different cultural contexts is lacking.
**Impact of Demographic Factors**	Found that female students exhibit higher BW behavior compared to male students. <br> − Determined that BW behaviors decrease with increasing age and education level.	Long-term effects of BW behaviors across different age groups need further investigation.
**Impact of Employment Status**	- Discovered that students who have never worked exhibit more BW behavior than those who have worked or are currently working.	Deeper investigation into the effects of different job and employment statuses on BW is needed.
**Platform Preferences and BW**	Students who prefer Puhu TV exhibit higher BW behavior than those who prefer Netflix and Amazon Prime.	Further research with larger samples on the impact of different digital platforms on viewing habits is required.

What sets this study apart from others is its focus on examining the determinants of binge-watching (BW) behaviors among university students within the cultural and demographic context of Turkey. Unlike previous studies, this research provides a detailed analysis of how BW behaviors vary according to demographic factors such as gender, age, education level, and employment status. Notably, the findings reveal that female students show a higher tendency toward BW than male students, BW behavior decreases with age and education level, and students who have never worked exhibit higher BW tendencies. Although the study showed a slight decrease in BW attitudes/behaviors as age increases, the limited age range of the university student sample suggests that further research is needed to explore this relationship across a broader age spectrum. These results offer a unique cultural perspective on media consumption habits among university students in Turkey.

This study sought to measure the binge-watching habits of university students on digital platforms and to identify how these habits differ according to the demographic characteristics of the participants, addressing four main research questions. Researchers first tackled questions in this context to identify participants' motivations for watching internet series. The responses from university students revealed that they engage in this behavior driven more by positive motivations; negative emotions such as depressive reasons, desire for escapism, and loneliness were emphasized less by the participants. The researchers determined that the motivations to which participants responded most positively were accessibility, entertainment, information seeking, relaxation, and social interaction. Moreover, the study utilized central tendency statistics related to participants' motivations for watching internet series to determine the level of importance they assign to different motivations. These analyses concluded that participants attribute a "high" level of importance to the relaxation/escape dimension, while entertainment, information seeking, and social interaction motivations are considered of "medium" importance.

The data from this study corroborate the findings of various previous research, emphasizing the significance of positive motivations such as accessibility [32,46,55], entertainment [15,32,63], information seeking [16,21,62,69,70] relaxation [15,31,32,46,63,71], and social interaction [31,63] in driving binge-watching behavior. These results validate the literature highlighting the prominence of hedonic motives in watching internet series [31,72].

The results related to the second research question of the study also support previous literature [73,74] showing that university students' attitudes/behaviors towards binge-watching (BW) are "high." Research specific to Turkey has shown that the trend towards BW among young people has increased, especially since Netflix began broadcasting as Netflix Turkey in 2016 [37,75]. The launch and rapid subscription growth of national and international online streaming platforms such as Amazon Prime, Blu TV, Puhu TV, and Exxen influence this increase. Furthermore, as various studies have shown, the COVID-19 pandemic's stay-at-home period has also significantly increased watching frequency and spread BW behavior [13,56,61,76].

When we examine the responses to which participants have given the most positive feedback, we understand that the most significant factor triggering BW on digital platforms is the ability to watch content without interruption from advertisements. This highlights the importance of an uninterrupted viewing experience as a critical driver for BW, suggesting that viewers highly value seamless and engaging content consumption, which digital platforms can provide. This insight is crucial for content providers and platform designers in optimizing user experience to cater to this preference for uninterrupted viewing. When analyzing the responses that participants most positively engaged with, it becomes clear that the ability to watch content on digital platforms without interruption by advertisements is identified as the most crucial factor leading to binge-watching (BW). These findings align with the research of Damratoski et al. [77] and Schweidel & Moe [6]. Compared to internet series, television series episodes in Turkey are significantly longer, and the commercial breaks on national television channels are frequent and lengthy. Consequently, for viewers accustomed to traditional broadcasting, the uninterrupted and thus perceived shorter episodes offered on online platforms are appealing. In conclusion, for viewers accustomed to traditional broadcasting, the uninterrupted and thus perceived shorter episodes offered on online platforms are appealing. This finding aligns with the results of our study, which showed that one of the primary motivations behind university students' binge-watching (BW) behavior is the ability to consume content without interruptions from advertisements. Participants responded most positively to the ability to watch content continuously, without breaks. The time required to watch a single episode of a series on television can allow for watching an entire season of a mini-series on digital platforms. This situation becomes understandable, especially for the new generation accustomed to short, ad-free, and quickly consumed visual content on YouTube and social media platforms. The second significant factor leading to BW, as shown by Devasagayam [78]and Flayelle et al. [63], is immersion, characterized by the viewer's absorption into the show. Reasons such as narrative flow, identification with characters, and the story's seamless continuation across episodes [54,62,63] can get viewers absorbed into the show and unwittingly engaging in long viewing sessions.

Current media studies classify relaxation as an intrinsic stimulus for media consumers [70,79]. When looking at the binge-watching (BW) items that participants responded to most positively, it is evident that avoiding interruption by advertisements and being deeply involved in the narrative for relaxation purposes are significant motivations in this regard. These two primary determinants largely stem from the structure of online streaming platforms and offer clues about why young people are moving away from watching television to consuming content online. Moreover, the emphasis on immersion suggests a tendency to lose control over the viewing experience. While these data support the role of hedonic motivations such as pleasure and entertainment in encouraging binge-watching, as highlighted in other research [21,31,47,55,62], the study also validates literature that indicates quite eudaemonic motivations like personal enrichment or information seeking are determinants in the emergence of BW behavior [16,21,62]. When examining the items participants engaged with at lower levels, concerns about body image and physical and psychological discomforts associated with binge-watching (BW) stand out. Previous research has also linked BW with concerns related to body image and health [80,81], as well as physical and psychological discomforts [75].

Investigations into how BW attitudes/behaviors vary according to participants' demographic characteristics have been conducted across variables such as gender, age, duration of education, employment status, and monthly expenditures. The results indicate that gender is a significant predictor of BW attitude levels. Female students have significantly higher BW attitudes than male students, corroborating general literature findings [82]. The higher BW attitude levels among women than men could be influenced by gender-based social expectations and roles, which affect preferences for leisure activities differently. Additionally, considering studies showing that women exhibit higher levels of attitudes toward emotional eating than men [83], the significantly higher BW behavior among women becomes more understandable. The research findings indicate a trend of decreasing binge-watching (BW) behavior with increasing age and duration of university education. Students aged 18–23 exhibit more BW tendencies than those aged 24 and above; similarly, students in their first four years of university are more inclined towards BW than those studying longer. The lower BW attitudes among university students and individuals over 24 compared to adolescents can be interpreted as the importance of academic success and time management. These results support findings from different studies in the literature [75,84].

Participants' BW attitude levels also significantly differ based on their employment status. Interestingly, participants who have previously worked part-time have significantly higher BW attitude levels than those who have never worked, are not currently working, or are currently working full-time. Part-time working students might perceive themselves as having less leisure time due to their experiences balancing study, work, and social life. This could lead them to seek more intense entertainment during their limited free time, thus showing a greater inclination towards BW. No significant relationship was found between participants' BW attitude levels and their monthly spending amounts. This suggests that financial expenditure on entertainment or other areas does not directly influence attitudes towards BW. As anticipated, the analysis results show that the frequency of watching series online increases with the tendency towards binge-watching (BW) behavior. This situation highlights the relationship between BW and the usage habits of streaming platforms. Measures such as offering entire seasons of serialized content for binge-watching, interface design, absence of viewing flow disruptions, and algorithmic recommendations contribute significantly to making BW a norm of viewing behavior on streaming platforms. Thus, every individual who begins consuming content on these platforms potentially becomes a marathon viewer, with the frequency of content consumption increasing the occurrence rate of this behavior. Employment status significantly influences BW behavior. Participants who have never worked may have more free time and fewer responsibilities, leading to greater opportunities for prolonged viewing sessions. The absence of structured daily routines and work-related commitments in this group likely contributes to increased BW behavior. As age and education level increase, individuals typically acquire more responsibilities. However, those who have never worked still show higher BW behavior, possibly due to less developed time management skills and lack of routine. Employed individuals might balance leisure activities better and have less time for binge-watching due to structured routines and work-related stress, which can limit their viewing time. The interaction between age, education level, and employment status is critical in understanding BW behavior. Older individuals or those with higher education levels who have never worked may still binge-watch extensively due to their available time and intellectual curiosity.

In conclusion, the research findings reveal that participants' BW attitude levels significantly differ based on their preferred platforms. Participants who prefer Puhu TV, a streaming platform unique to Turkey, exhibit higher BW behavior rates than those who prefer Netflix and Amazon Prime. This difference may be due to Puhu TV's offering of free access to series and possibly being culturally closer to university students. Other research examining how digital platforms differentiate viewing styles will likely provide significant data for a more detailed explanation.

## 6 Practical implications and recommendations

The findings of this study reveal several key insights into university students' binge-watching (BW) behaviors, which have practical implications. First, the significant correlation between entertainment motivation and BW behaviors suggests that streaming platforms should consider enhancing entertainment features that cater to students' preferences, such as personalized content recommendations and uninterrupted viewing experiences. Since students are particularly motivated by ease of access and the ability to watch without advertisements, streaming services can develop features that minimize interruptions and improve user engagement. Moreover, the finding that female students exhibit higher BW tendencies compared to male students indicates a potential need for gender-specific strategies. Awareness programs at universities can target female students to address potential negative outcomes of BW, such as physical inactivity and sleep disruptions, while promoting balanced viewing habits. Additionally, as the study showed a slight decrease in BW behavior with increasing age and education level, younger students or those in early stages of their education could benefit from time management workshops. These interventions can help students develop better media consumption habits and balance their academic responsibilities with leisure activities.

This study reveals the prevalence of binge-watching (BW) behavior among university students and its association with demographic characteristics. The findings provide several practical implications and guidance to avoid negative effects:

Universities can develop awareness programs to help students avoid the negative effects of BW behavior. These programs should promote healthy viewing habits and time management. Students should be informed about the potential negative impacts of binge-watching on academic performance, sleep patterns, and physical health.

Families should monitor their children's media consumption habits and help them establish balanced viewing times. Additionally, offering alternative entertainment and relaxation activities can help mitigate the negative effects of excessive viewing. Digital content platforms should provide tools to help users develop healthy viewing habits. For example, tracking viewing times and sending reminders can help users keep their binge-watching behavior under control.

## 7 Conclusions, limitations, and recommendations for future research

The research findings indicate that students' primary motivations to watch internet series are easy access, entertainment, and the desire to interact with different people. As age and education level increase, the attitude towards binge-watching (BW) decreases. At the same time, participants who have never worked exhibit more BW behavior than those who have worked or are currently working. However, it was found that income level does not significantly affect BW behavior. The study concludes that BW is widespread among university students and can be associated differently with various demographic characteristics. Awareness of BW's positive and negative aspects is essential for better understanding and managing this behavior. The study includes several limitations that highlight the need for further research to question the findings of the current research on understanding BW. Conducted solely among a segment of university students in Turkey, the study's sample is limited to students from Selcuk University. This indicates that the findings may not be generalized to all university students in Turkey. Future studies should include participants from diverse cultural and geographical backgrounds to understand how BW behavior interacts with cultural dynamics. The voluntary basis of participants' inclusion in the study suggests that students who do not exhibit BW behavior may have been less interested in participating. Therefore, new research should be conducted on different sample groups, and the obtained data should be tested, which could strengthen the generalization of the findings. Additionally, conducting studies in various cultural contexts to understand the interaction of BW behavior with cultural dynamics would also be beneficial.

The current study, based on data collected through a questionnaire, acknowledges that it may not always be possible for participants to objectively assess their behaviors and motivations. Therefore, future research could incorporate different data collection methods, such as behavioral tracking technologies and biometric measurements, alongside participants' self-reports. These methods could contribute to more accurately evaluating binge-watching (BW) behavior. Additionally, qualitative research methods could be employed to deeply examine participants' BW behaviors, motivations, and the effects of this behavior on their lives, which would aid in gaining a more comprehensive understanding of the BW phenomenon. The study's cross-sectional research design does not allow for determining causality between BW behaviors and motivations. Longitudinal studies should be conducted to examine the long-term effects and changes in BW behavior, which is crucial for better understanding causal relationships. Lastly, although the current study concluded that income level does not significantly affect BW behavior, more research is needed to delve deeper into this relationship. Comprehensive research identifying existential factors that could affect BW behavior would enrich our understanding of how various socio-economic and personal factors influence media consumption patterns.

Additionally, future research should compare BW behaviors among students who watch series through different platforms (internet, television, etc.) and examine the various aspects of BW behavior, including frequency, duration, and genre preferences. Understanding the positive and negative outcomes of BW behavior is also important, and intervention programs could be developed to help manage excessive BW behavior.

## Data availability statement

The data associated with this study has not been deposited into a publicly available repository. However, the data will be made available on request. Researchers interested in accessing the data for the purpose of evaluating the findings, building on the work, or increasing trust in the article are encouraged to contact the corresponding author.

## CRediT authorship contribution statement

**Murat Aytas:** Writing – review & editing, Writing – original draft, Methodology, Conceptualization. **Ismail Berkay Topatan:** Writing – original draft, Investigation, Conceptualization.

## Ethical considerations

The data was processed according to the principles of the Helsinki Declaration. The study was approved by the Selcuk University Research Ethics Committee (Approval ID: January 11, 2021 -E.100851).

## Funding

This research did not receive any specific funding.

## Declaration of competing interest

The authors declare the following financial interests/personal relationships which may be considered as potential competing interests:Murat Aytas reports administrative support was provided by Selçuk University. If there are other authors, they declare that they have no known competing financial interests or personal relationships that could have appeared to influence the work reported in this paper.
